# Manipulating perceptual parameters in a continuous performance task

**DOI:** 10.3758/s13428-017-0877-7

**Published:** 2017-03-31

**Authors:** Nir Shalev, Glyn Humphreys, Nele Demeyere

**Affiliations:** Cognitive Neuropsychology Centre, Department of Experimental Psychology, University of Oxford, Oxford, OX1 3UD UK

**Keywords:** Sustained attention, Alertness, Continuous performance task, CPT, Stroke, Temporal attention, Attention, Ageing, Chronic stroke, Methods

## Abstract

Sustained attention (SA) is among the most studied faculties of human cognition, and thought to be crucial for many aspects of behavior. Measuring SA often relies on performance on a continuous, low-demanding task. Such continuous performance tasks (CPTs) have many variations, and sustained attention is typically estimated based on variability in reaction times. While relying on reaction times may be useful in some cases, it can pose a challenge when working with clinical populations. To increase interpersonal variability in task parameters that do not rely on speed, researchers have increased demands for memory and response inhibition. These approaches, however, may be confounded when used to assess populations that suffer from multiple cognitive deficits. In the current study, we propose a new approach for increasing task variability by increasing the attentional demands. In order to do so, we created a new variation of a CPT – a masked version, where inattention is more likely to cause misidentifying a target. After establishing that masking indeed decreases target detection, we further investigated which task parameter may influence response biases. To do so, we contrasted two versions of the CPT with different target/distractor ratio. We then established how perceptual parameters can be controlled independently in a CPT. Following the experimental manipulations, we tested the MCCPT with aging controls and chronic stroke patients to assure the task can be used with target populations. The results confirm the MCCPT as a task providing high sensitivity without relying on reaction speed, and feasible for patients.

## Introduction

As our environment is constantly changing over time, we must detect those changes and act accordingly. This is why the ability to remain vigilant, or sustain attention over time, is a prerequisite for almost every aspect of adaptive behavior. Acknowledging the importance of the ability to sustain attention, researchers have studied individual performance over time from the earliest days of contemporary cognitive research (e.g., Mackworth, 1948). There is ample evidence demonstrating how sustained attention is crucial across the life span. Difficulties in sustaining attention are correlated with learning, behavioral, and emotional difficulties in adolescence (e.g., Shalev et al., 2015), with professional development (e.g., Kalechstein, Newton, & van Gorp, 2003), driving (e.g., Schmidt et al., 2009), and more. Sustained attention failures are also affiliated with various psychiatric disorders, such as sub-groups of attentional deficit hyperactive disorder (e.g., Tsal et al., 2005), autism (Garretson, 1990), learning difficulties (Richards et al., 1990), and schizophrenia and affective disorders (Liu et al., 2002). Another clinical group who often suffer from impaired sustained attention are patients with brain lesions (e.g., Hyndman & Ashburn, 2003; Robertson et al., 1997a). Within this group, beyond the everyday implications we discussed so far, sustained attention has been found to be an important factor in recovering from other cognitive syndromes caused by brain injuries, such as motor problems (Robertson et al., 1997a) or unilateral neglect (Robertson et al., 1995).

Typically, sustained attention is assessed based on variations of the continuous performance test (CPT). In a CPT, participants are required to maintain their concentration level over time during a relatively simple (and inherently boring) task: the identification of a pre-specified target within a continuous stream of distractors (e.g., Conners & Staff, 2000). One type of outcome measure is based on accuracy: the number of omission (“miss”) and commission (“false-alarm”) errors. However, in most tasks these variables do not provide enough variability between individuals due to a ceiling effect in accuracy (Halperin et al., 1991; Robertson et al., 1997b; Sarter et al., 2001). Therefore, the more typical outcome measure of most CPT variations is the variability of reaction time (RT), supposed to represent the overall stability of performance. The lack of discrete outcome measures, however, such as the number of omission errors as an index for task performance, may pose a problem when applied to some clinical populations due to possible motor confounds when assessing RTs with such groups (e.g., Ada et al., 1996; McCrea & Eng, 2005).

One way to address the issue of ceiling performance was suggested by Robertson and his colleagues (1997b), introducing the SART task. In the SART task, participants have to respond to all distractors by pressing a button – and to withhold their response whenever identifying a target. Indeed, this task configuration significantly increased the number of commission errors (false alarms), allowing high sensitivity in a non-RT based measurement. A different approach is based on increasing memory demands, such as in the case of the CPT-AX (e.g., Chen & Faraone, 2000). In the CPT-AX participants have to respond to a target – the letter “X” – only if it appeared after the letter “A”. Therefore, they have to store in their memory the identity of the target and the pre-target stimulus. Other examples of manipulations researchers have used in order to increase task sensitivity include stimulus degradation (e.g., Parasuraman, Mutter, & Molloy, 1991) and attentional load (Shalev et al., 2011). Nevertheless, it seems like all the different variations suffer from the same trade-off: either the task relies on RT-based outcome measurements, or it is at risk of being confounded by unrelated cognitive constructs. For instance, many patients with brain lesions suffer from response inhibition problems (e.g., Aron et al., 2003); since the outcome measure in the SART is based on failing to inhibit responses to targets, it is difficult to imagine how one can differentiate between poor attention and poor inhibition when applied to patients (also see Ballard, 2001). A similar argument can be made with poor memory and performance on the CPT-AX (for more about the involvement of memory in CPT-AX see Lee & Park, 2006).

A recent solution for the trade-off between retrieving a discrete outcome measure based on accuracy while avoiding confounds was recently suggested by O’Connell and his colleagues (2009). In their task, the continuous temporal expectation task (CTET), participants observed a continuous stream of squared patterned stimuli flickering on the screen and alternating its orientation every 800 ms or 1,120 ms. When participants observed a stimulus which remained for 1,120 ms (the longer exposure time), this was the target to respond to. In this task, the accuracy rate was very low compared to other paradigms, and reached an average of 67% (O’Connell et al., 2009) in healthy young participants. Another key benefit of the CTET, besides its evident sensitivity, is that the task is made up from a continuous stream of information in the same spatial location. The presence of a constant stimulus may help to prevent a possible confound of spatial orienting of attention by target onset. When performing a normal CPT, arguably several attentional processes are involved in the target discrimination: attentional shift and engagement, followed by target selection. This is because of the way most CPTs were designed, with an abrupt onset of targets and distractors over a blank screen (or a screen with a fixation point). This way, the mere appearance of either a target or a distractor acts as an exogenous cue, causing attentional capturing in a bottom-up manner. In other words, when the target or distractor suddenly appears, the abrupt onset attracts attention. Only after attention is oriented and engaged to the stimulus, the processes of target discrimination are initiated. The CTET paradigm avoids this confound with a continuous presentation at the center of the screen throughout the task. This way there is no abrupt onset, and therefore less involvement of orienting mechanisms of any kind.

Although the CTET hereby resolved some important issues in measuring sustained attention, when it comes to clinical populations there are some shortcomings. First, the low accuracy rate received with neurologically normal participants may mean that this task is too difficult for some populations. Second, and more important, when moving from the well-established method of visual target discrimination to temporal judgment, there is a risk of recruiting other unrelated mechanisms that could be impaired independently. Some studies have already observed specific problems in temporal judgment after brain injury (e.g., Lackner & Teuber, 1973; Robin, Tranel, & Damasio (1990). Therefore, in the case of the CTET, some performances may be relying on several mechanisms that are not necessarily related to sustained attention, and may confound any observation in cases of working with patients.

The following study aimed to establish a novel approach for assessing sustained attention. We modified a CPT task to resolve some of the major challenges we raised hitherto: while maintaining the well-established goal of a CPT task, it should facilitate a higher error rate outcome measure, even in a healthy population. This will allow the assessment of sustained attention without relying solely on RT. At the same time, the task should be suitable for various populations (simple, not too difficult); it should not require a high memory capacity; it should not be dependent on unimpaired inhibitory control mechanisms; and it should be continuous, in order to avoid the abrupt onset of targets and distractors. With these constraints in mind, we aimed to increase task performance variability based on individual differences in attention, rather than memory, motor skills, or inhibitory control. In particular, when working with clinical populations, there is the danger that other cognitive impairments, unrelated to attention, could camouflage the individual sustained attention capacity when assessed using a cognitive task. Importantly, our aim in this particular study was to develop an assessment tool – and not to test individual differences in sustained attention. Therefore, our analysis does not focus on individual profiles of performance, and instead aims solely to establish increasing variability and sensitivity in error rates.

## Experiment 1: Continuous performance task with/without masking

Experiment 1 employed a novel CPT paradigm, which was designed to derive interpersonal variability in accuracy-based measures, even in a young, healthy population. The new test aimed to create a more inclusive assessment, avoiding the use of speeded responses, which in various clinical populations may confound the measure (e.g., Ada et al., 1996; McCrea & Eng, 2005). Typically, young healthy participants perform at ceiling in CPT tasks, making almost no errors (Halperin et al., 1991), and the standard deviation of RTs (RTSD) is used to derive a more sensitive measure of sustained attention in these high-performing groups (e.g., Shalev et al., 2011). Our paradigm was aimed to enhance the number of task errors, bypassing the need to rely on RT-based outcome measures to assess sustained attention performance.

In order to achieve this, in our version the CPT the targets and distractors are continually masked (pre- and post-mask). Following previous work showing greater task sensitivity with a conjunctive set of stimuli (e.g., Shalev et al., 2011; Tsal et al., 2005), the target is defined by a conjunction of features (color and shape), and the distractors could share these features. We introduced the new masked conjunctive continuous performance test (MCCPT) to a young healthy control sample. Experiment 1 compared performance in our new MCCPT task with a non-masked version of the same task (CCPT).

We had two main reasons to believe that adding a mask to the CPT paradigm would help us create a clearer and more inclusive measure of sustained attention. First, by using a mask in our newly developed task, we avoid the abrupt onset of targets and distractors. During the MCCPT task, there is a continuous stream of visual stimuli which decreases the spatial cuing to a minimum and requires a continuous engagement at the same location on the screen in a goal-directed manner.

Second, by using a mask we degraded the stimuli. This meant that if participants failed to attend to the shape while it was presented, they would simply miss it and make an omission error. The perceptual degradation of the stimulus could be compensated by attention, based on the familiar notion that engaged attention enhances perception (e.g., Muller & Humphreys, 1991; Posner, 1980). Hence, our MCCPT allows us to ensure participants will use their attentional system to identify the target and discriminate it from the distractors. Importantly, as opposed to previous paradigms where perceptual degradation was used (e.g., Parasuraman, Mutter, & Molloy, 1991), in our task we do not interfere with the stimuli itself. Therefore, our degradation can be compensated by using attention, as opposed to cases where targets are blurred and may be missed due to perceptual limitations.

The interplay between visual masking and attention has been extensively studied in the past. Within the attention literature, attending a stimulus is thought to reduce the effect of masking (Enns & Di Lollo, 1997). Another experimental tradition, closely related to attention, in which masking is often used is in the study of visual short term memory (VSTM) consolidation. Here a visual mask is deployed to interfere with iconic memory representation (e.g., Gegenfurtner & Sperling, 1993; Shibuya & Bundesen, 1988). Within this context, researchers have found that the process of consolidating items from their fragile iconic representation into VSTM occurs within an early timeframe following stimulus exposure, ranging between 30 ms (Shibuya & Bundesen, 1988) and 50 ms (e.g., Vogel, Woodman, & Luck, 2006). An integrative perspective of VSTM encoding and visual attention has been described as part of the theory of visual attention (TVA). The TVA is a mathematical formalization of the “biased competition” account of visual attention (Duncan & Desimone, 1995), where visual categorizations ascribing features to objects compete to be encoded into a limited capacity VSTM. The categorization of a visual element is accomplished once it has been encoded to VSTM. In line with this perspective, we consider attention to be the mechanism that can prioritize elements to be stored in VSTM, and in the context of our current study, attention would be the mechanism which needs to be deployed efficiently as the target appears, in order to be encoded before the masking will appear and erase iconic traces. In addition to this, we also ensured a sufficient stimulus exposure time to formulate a VSTM representation.

If indeed adding the mask increases error rate, this will lead to a clear hypothesis about the relation between the outcome measures: RTSD and number of omission errors should be correlated. The reason for this hypothesis is our notion that they both reflect the same construct: “attentional disconnections,” or “temporal inattention.” Importantly, these variables reflect mathematically independent values: omission errors total the number of errors where participants did not respond to the target, whereas RTSD relies on RTs for correct target identification only. As opposed to omission errors, the case of comissions (“false alarms”) is a little bit trickier. While comissions can result from an “attentional slippage,” they may also result from a failure in inhibiting prepotent responses, as often observed in go/no-go tasks (e.g., Nieuwenhuis et al., 2003). Therefore, any correlation we might find between commissions and RTSD should be smaller compared to omissions and RTSD.

One way to incorporate the two error types while controlling for the involvement of response inhibition mechanisms is by using parameters derived from the signal detection theory (SDT; Green & Swets, 1966). In SDT, the perceptual sensitivity parameter (d’) incorporates the two error types: omissions and commissions. Another parameter derived from SDT is the criteria parameter (β), which indicates the bias towards a perceptual decision: participants can be biased either towards missing targets (omissions) or towards responding to distractors (false alarms, or commissions). Here, we suggest using the SDT parameters as two different task indices: d’ as the marker of task performance, and β as a control for the dominant error type. By maintaining the β value either at zero or at a positive value, we assure that most of the errors committed were omission errors. As we will further verify in Experiment 2, the main characteristic of a task demanding response inhibition is a negative β value.

Performance in our new task was compared to performance in a variation of the CPT: the conjunctive CPT (CCPT; Tsal, Shalev, & Mevorach, 2005). In this variation, participants are required to identify a target shape and ignore distractors, some of which have conjunctive features (either the same color or same shape). The use of conjunctive features for distractors was found to increase demands for attention while maintaining high task reliability (Shalev et al., 2011). In our case, the use of the CCPT as a control allowed us to investigate the influence of only one task factor – adding the mask inbetween stimuli. In order to make it suitable for various populations, other than adding a mask, the properties of the CCPT were preserved as in Tsal et al. (2005): it is based on shapes and not letters or numbers, and does not require holding more than one target in memory.

## Method

### Participants

Twenty-two naive volunteers participated in this experiment (ten female). They were recruited through an online research participation system at the University of Oxford. All had normal/corrected-to-normal eyesight and were right-handed (mean age 28.4 years, SD 4.95). They were compensated for their time (payment of £10 per hour, inclusive of travel expenses).

### Apparatus

A PC with Intel i7 processor and a dedicated 2GB AMD video card was used for displaying stimuli and recording data. The task was generated using NBS presentation software (Neurobehavioral systems, Albany, CA, USA). The stimuli were presented on a ViewSonic V3D245 LED monitor, with screen resolution of 1,080X1,920 and a screen refresh rate set at 100 Hz allowing display times varied in gaps of 10 ms. All stimuli were preloaded to memory using the presentation software, to guarantee minimal temporal noise.

### Task 1: MCCPT sustained attention

**Fig. 1:**
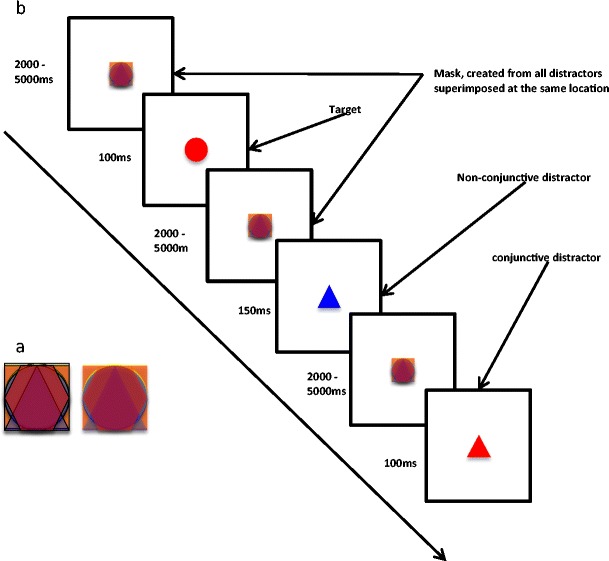
(**a**) The two alternating masks; (**b**) the Masked CCPT sustained attention schematic outline. Values are in ms

A colored mask (*Mask*), comprised of four superimposed figures in different colors (square, triangle, circle, and hexagon) appeared at the center of the screen. The total size of the mask occupied 3° visual angle. In order to avoid habituation effects, we generated minor movements to the Mask. The movement was generated by alternating every 10–20 ms between two mask-images, one of which had thicker outlines for the superimposed figures (the two alternating mask images are illustrated in Fig. 1a). The mask appeared at the center of the screen and disappeared only when it was replaced by either a target or a distractor shape for 100 ms; the mask then reappeared immediately, generating pre- and post-masking of each target or distractor. The target shape was a red circle, and distractor stimuli were either similar in color to the target (red hexagon and red triangle), similar in shape (blue circle and red circle), or completely different (yellow and blue hexagon). All distractor types appeared in an equal distribution. All distractors and target shapes appeared at the center of the screen and circumscribed a square of 3° visual angle. The inter-stimulus interval was jittered, randomly between 2,000 and 5,000 ms (see Fig. 1b for a schematic outline of the experimental procedure). Participants were told that the static shape that appeared at the center of the screen (the mask) would be replaced every few seconds with another shape which would appear only briefly. The task was to press as fast as possible whenever they recognized a *red circle* at the center of the screen. They were instructed to do nothing when they saw any other shape.

### Task 2: Non-masked CCPT

Task 2 was identical to Task 1, apart from removing the masking condition. There was no mask present at any time. Participants simply focused on the center of the screen and indicated when they identified a target. All other parameters remained the same.

#### Procedure

All participants performed both tasks, with a short break in-between. The order of administration was balanced across the group. Each task started with a short practice block (15 trials), and the experimenter monitored participants’ responses at this stage to ensure the instructions were clear. For each task, after finishing the practice, the participants performed the whole session without any break until the task terminated after approximately 13 min. Each task was comprised of 240 trials. The target appeared on 80 trials (33% *target)*; and there were 160 *distractor* trials (66%*)* in which one of six possible distractors appeared on the screen in a randomized order. A distractor was either a *color-conjunctive-distractor*, where it had the same color as the target (22%); a *shape-conjunctive-distractor*, where it had the same shape (22%); or a *non-conjunctive-distractor* where it differed from the target in both shape and color (22%).

#### Statistical analysis

For each participant, we extracted data about the correct reports of targets, the number of omission errors and the number of commission errors, as well as RTs. These measures allowed us to calculate individual performance according to multiple indices : (a) the standard deviation of reaction time; (b) sensitivity, or the discriminability of the target from distractors (d’), in accordance with the signal detection theory (SDT); and (c) the criterion for the perceptual decision (β) (also based on SDT).

Whereas the ability to discriminate target from distractor (d’) incorporates the two error types – commissions and omissions, the criteria (β) provides a measure of the balance between error types: a positive value means a higher tendency towards omission errors, and vice versa (when β value is zero, there is no bias towards any particular error type). The β parameter will be used as a measure for understanding whether the task facilitates inattention-based errors or inhibition-based errors. As we suggested earlier, commission errors can be resulted not only from instances of temporal inattention, but also from a failure in response inhibition. By measuring the β parameter, we attempt to control for this by estimating which error type is more dominant: presumably, when stressing one’s sustained attention, we expect to see either no bias or a bias towards omission errors. Conversely, if we discover a bias towards commission errors, perhaps our task also involves high requirements for response inhibition. This working assumption will be tested separately in Experiment 2.

We compared all the outcome measures to test for consistency within and between the masking and no-masking versions of the tasks, and in order to determine whether the use of masking indeed increases the sensitivity of our measurements, as we predicted.

## Results

First, we used multiple methods to confirm the reliability of both the MCCPT and CCPT task. Prior to the analysis, we removed one participant whose accuracy performance in both tasks was below three standard deviations compared to the rest of the group.

### MCCPT

Based on previous studies using CPT, and considering the fact that we tested normal young participants with no motor limitations, we started the process of task validation by assessing reaction time related variables. In order to test for internal consistency in RTs and the standard deviation of reaction time (RTSD), we split the data into four quartiles and calculated for each the RT and RTSD for correct target identifications. Then, we used Cronbach’s alpha test for the four quartiles which yielded a high consistency of .948 for RT and .843 for RTSD.

After verifying internal consistency, we tested for correlations between error types and RT related measures. Because our group was smaller than 30 and our calculations are based on discrete variables, we calculated a correlation using Spearman’s rho test for non-parametric correlation. In accordance with our initial hypothesis, the number of omission errors was significantly correlated with RTSD (Spearman’s rho (21)=.74; p<.001). A similar high correlation was found between our main construct – target detection d’ – and RTSD (Spearman’s rho (21)=-.69; p=.001), demonstrating that a lower ability to discriminate target was linearly related to a high variability in reaction times. The correlation between the number of commission errors and RTSD was not significant (Spearman’s rho (21)=.30; p=.18).

### CCPT

The analysis of CCPT data was carried out following the same procedures as for the MCCPT task. When testing for task consistency, as we did for the MCCPT task, we split the responses into four quartiles and averaged RT and RTSD for each. Cronbach’s alpha test for the four quartiles yielded a high consistency of .897 for RT and .794 for RTSD.

We also tested for correlations between RTSD and error type: once again, as we hypothesized, omission errors were significantly correlated with RTSD (r(21)=.62; p=.002), and no significant correlation was found between commission errors and RTSD (r(21)=.03; p=.99)

#### Comparing and cross-validating CCPT and MCCPT sustained attention

For the purpose of cross-validating the tasks, we assessed the correlation between individual performance on each task. We found a significant correlation between the RTSD measure of the masked and unmasked CPT (r(21)=.75; p<.001). A similar high correlation was observed for sensitivity (d’) (Spearman’s rho (21)=.68; p=.001) and for the criteria (β) (Spearman’s rho (21)=.77; p<.001).

After this confirmation that our new MCCPT indeed reliably assessed the same parameters as the CCPT, we carried out a series of direct comparisons between the two. A repeated measures t-test revealed a significantly higher RTSD in MCCPT (141 ms) compared to CCPT (114 ms) (t(20)=2.18; p=.04; 95% CI [1.17–50.74]). We also found that sensitivity to target (d’) was significantly higher in CCPT (d’=4.1) compared to MCCPT (d’=3.64) (t(20)=-2.76; p=.012; 95% CI [-.80 to -.11]), and suggests that participants had more difficulty in differentiating the target from the distractors in the masking condition. We performed a further analysis of target discriminability by comparing the number of commission errors for conjunctive distractors (e.g., distractors sharing the same color or shape as the target) compared to non-conjunctive distractors. Our comparison showed a significantly higher percentage of errors for conjunctive distractors (t(21)=4.75; p<.001). The criteria variable (β) was significantly higher in MCCPT (β=0.274) compared to CCPT (β=0.089) (t(20)=4.46; p<.001), 95% CI [.098–.270]) demonstrating a higher bias towards omission errors in the masked condition. More important, in the masked condition the bias parameter was significantly larger than zero (t(20)=3.98; p=.001; 95% CI [.130–.417]), and in the non-masked it was not significantly different from zero (p=.12). Therefore, in accordance with our hypothesis, in these particular task settings participants had either a dominancy of omission errors (masked task)– or no dominancy of any specific error type (non-masked MCPT).

## Interim discussion

Our results clearly demonstrated how adding a mask in between targets on a sustained attention task increased the general task sensitivity: participants had a higher RTSD, showed an increased bias towards omissions, and had a lowered target discriminability (d’). Earlier we argued that the bias parameter could be used to control for the involvement of inhibitory mechanisms. This is based on the assumption that in a task that has high demands for inhibitory control, we should observe a higher proportion of commission targets (resulting from failure to inhibit prepotent responses; on measuring response inhibition see e.g., Aron & Poldrack, 2005).

One potential variable affecting the proportion of commission errors, and therefore also the bias direction, is the target frequency: increasing the target frequency should increase the number of responses, which may therefore increase the demands for cognitive control to withhold the prepotent responses in case of irrelevant distractors (see also e.g., Swick, Ashley, & Turken, 2008). Furthermore, we demonstrated how the masked sustained attention task configuration creates a positive β value, which reflects a general bias towards missing targets. If increasing the number of targets increases the requirement for inhibitory control, we should observe an increase in commission errors resulting in a modulation of the bias parameters towards negative values (i.e., bias towards false alarms).

A better understanding of continuous performance tasks is crucial for improved diagnostics of attentional disorders. There is currently no single convention for an optimal outcome measure for sustained attention, and in many cases commission errors, omission errors, and RTSD are used interchangeably. Here we attempt to incorporate the different error types using d’, while assuring that there is no bias towards commissions. Experiment 2 aimed to investigate if there is a difference in the pattern of performance when we re-design the *sustained attention* task to act as a *go/no-go* task*.* The only difference between this Experiment 1 and Experiment 2 is the target-distractor ratio: in a go/no-go task, it is customary to challenge participants with a high frequency of targets, to encourage more responses and more false alarms (Simmonds, Pekar, & Mostofsky, 2008). We used the same task design as in Experiment 1, but increased the target probability from 33% to 66%. We hypothesized this change will influence mostly the bias SDT parameter reflecting the more probable type of error (commissions vs. omissions).

### Experiment 2: Inhibitory control task with/without masking

We hypothesize that calibrating the CCPT and MCCPT into a *go-no/go* task only by increasing the proportion of targets will influence only the decisional criteria (β), which will become negative comparing to the sustained attention settings. Such a pattern of performance may reflect a greater involvement of inhibitory mechanisms, as false alarm errors are considered to be indices for lapses of inhibitory control in go/no-go tasks (e.g., Aron, Trevor, & Russell, 2004).

## Methods

### Participants

Twenty-two naive volunteers participated (nine female). All had normal/corrected-to-normal eyesight and were right-handed (mean age 27.8 years, SD 4.6). They were recruited and compensated for their time in the same way as in Experiment 1.

### Apparatus

The apparatus was the same as in Experiment 1.

#### Task 3: MCCPT Go/No-go

We repeated the same task configuration as in *Task 1: MCCPT*, only this time we inverted the target/distractor ratio, with 33% distractors and 66% targets.

#### Task 4: CCPT Go/No-go

We repeated the same task configuration as in *Task 2: CCPT*, only this time we inverted the target/distractor ratio, with 33% distractors and 66% targets.

### Statistical analysis

First, in order to compare the task to our findings from Experiment 1, we repeated the same procedure of task validation for the various outcome measures. Following this, we performed a direct comparison between the two experiments in order to pinpoint how performance on the different measures was influenced by the manipulation of the target-distractor ratio.

## Results

Consistent with our procedure from Experiment 1, task reliability was verified based on RT and RTSD over four quartiles. In the masked version (MCCPT), Cronbach’s alpha test for the four quartiles yielded a high consistency of .942 for RT and .731 for RTSD. In the non-masked version, Cronbach’s alpha test for the four quartiles yielded a high consistency of .935 for RT and .826 for RTSD,

### Comparing sustained attention and go/no-go tasks

Descriptive statistics appear in Table 1.

**Table 1:** Descriptive statistics – performance in masked and non-masked variations of the CCPT

	Go/No-go (masked)	Go/No-go (unmasked)	Sustained attention (masked)	Sustained attention (unmasked)
% Commissions (med, avg, SD)	5% ; 9% ; 8%	4%; 7%; 8%	2%; 3%; 2%	2%; 2%; 2%
% Omissions (med, avg, SD)	7%; 8%; 7%	1%; 2%; 3%	5%; 10%; 12%	1%; 5%; 8%
B (avg; SD)	-0.037; 0.310	-0.307; 0.286	0.274; 0.308	0.090; 0.247
D’ (avg; SD)	3.145; 0.885	4.025; 0.851	3.641; 0.846	4.102; 0.863

Next, we assessed which measures of task performance were influenced by changing the target-distractor ratio. We performed a series of direct comparisons between the two groups (Exp. 1 vs. Exp. 2, or the sustained attention and go/no-go manipulations), on each of the two conditions (masked vs. unmasked). For each of the critical variables, we carried out a mixed model 2x2 ANOVA, with the masked versus unmasked conditions as the within subjects factor and the low versus high target ratio as the grouping factor.

When applying the 2X2 ANOVA to the percentage of omission errors, we found a significant main effect for masking, with a higher percentage of omission errors whenever the stimuli were masked (*F*(1,39)=21.937, *p*<.001, η^2^=.360). We also observed a significant main effect for masking in the perceptual sensitivity parameter (d’) with higher perceptual sensitivity in the non-masked conditions (*F*(1,39)=22.817, *p*<.001, η^2^=.369). These observations are in line with our motivation for increasing task sensitivity by adding a mask. For the percentage of commission errors, we found a significant main effect for experimental condition (go/no-go vs. sustained attention), with more commission errors when we increased the number of targets (*F*(1,39)=14.766, *p*=.001, η^2^=.275). This is in line with our hypothesis that commission errors are sensitive to the involvement of inhibitory control (by increasing proportion of targets), whereas omission errors are not. For the criteria or response bias (β), we found a main effect for masking (*F*(1,39)=27.957, *p*<.001, η^2^=.418) with greater bias towards omitting targets in the masked condition (this is a replication of what we found in experiment 1). We also found a main effect for condition (*F*(1,39)=22.433, *p*<.001, η^2^=.365) with a greater bias towards omitting targets when distractors’ proportion increases. We did not observe any interaction between the proportion of targets and the use of masking (all p >.24). This is in line with our argument that the two task parameters influence the perceptual parameters independently.

## Interim discussion

In Experiment 2 we successfully established that increasing demands for response inhibition, by adjusting task parameters to a go/no-go task, changes the response bias towards a higher proportion of commissions errors. In other words, the response bias is the main task property that changes between a sustained attention task and a response inhibition task. Therefore, it can be considered as a control variable for the dominancy of inhibitory mechanisms.

At this point, we clarified that the perceptual sensitivity measure (d’) is decreased when we add a mask, and in Experiment 1 we demonstrated it also correlates with the more prevalent index of sustained attention – the RTSD. We can carefully hypothesize that d’ may be somehow influenced by sustained attention: potentially, a higher variability of attention may eventually lead to misidentification of targets. Indeed, it seems that with our perceptual manipulation (i.e., adding a mask and increasing perceptual demands) we caused these two correlated performance indices to change. We also noticed that the bias parameter (β) alternated between a bias for missing a target (in the sustained attention task settings) and committing a false alarm (in the Go/No-go task settings). The change in this parameter reflects the change in the pattern of errors: it is a way of representing the more frequent error type on each condition.

After establishing that the MCCPT indeed increased sensitivity in accuracy-based measures, and that performance patterns (as reflected in the bias parameter) were influenced by adjusting the appropriate target-distractor ratio, we aimed to investigate whether the task is feasible and informative in the target population. In Experiment 3, we used an adjusted version of the MCCPT with both a group of ageing individuals and chronic stroke survivors.

### Experiment 3: Testing older adults and clinical patients

In Experiment 3 we describe the accuracy based outcome measures of a sample of patients and older adults who performed the MCCPT (with a slight variation of the exposure times). A subset of this sample and the associated MCCPT data was previously described in an experimental paper focusing on the functional outcomes of impaired sustained attention (Shalev, Humphreys, & Demeyere, 2016).

## Methods

### Participants

The experimental group consisted of 75 participants, 42 of which were neurotypical adults (26 females; mean age 68.3 years; SD=8.1) and 33 were chronic stroke patients (11 females; mean age 63 years; SD=13.7). The groups did not differ significantly in their age or level of education. The clinical group consisted only of chronic stroke survivors, who had their stroke more than a year ago, and came as volunteers to the Oxford Cognitive Neuropsychology Centre; the patients varied in their lesion site and volume, and we did not exclude any of the available volunteers from taking part. (For a detailed description of the experimental settings, recruitment process, and the lesion sites of the majority of the clinical group, see Shalev, Humphreys, & Demeyere, 2016).

### Apparatus

The apparatus was the same as in Experiments 1 and 2.

### Task: MCCPT austained-attention

We repeated the same task configuration as in *Task 1: MCCPT*, only this time we extended the stimulus exposure time to 150 ms. We did so in order to ensure the task would be simple enough for all of the older participants and stroke survivors. We assessed in this experiment whether this relatively long exposure time still produces meaningful variability in our accuracy based outcome measures.

### Statistical analysis

We described the performance distribution of the two experimental groups, with an emphasis on accuracy-based outcome measures. Our goal was to make the task simple enough for the clinical population, while at the same time avoiding ceiling or floor effects in performance. We also compared the groups to see if they could be distinguished based on the task.

## Results

### Descriptive statistics

**Fig. 2:**
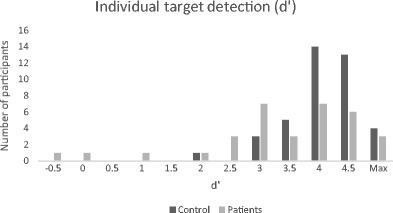
Individual target detection (patients and controls)

Task performance indices are described in Table 2. The distribution of individual detection rates in both groups is shown in Fig. 2.

**Table 2:** Descriptive statistics

	d' (mean; median; SD)	β (mean; median; SD)	% miss (mean; median; SD)	% false alarms (mean; median; SD)
Controls	4.28; 4.25; .55	.05; 0; .18	2.5; 1.6; .03	1.8; 1.2;.01
Patients	3.56; 3.77; 1.24	.03; 0.12; .36	7.8; 1.6; .1	7.5; 2.5; .13

Evidently, as with the younger individuals we have tested, the majority of the individuals did not reach ceiling performance even though we extended the stimulus exposure time to 150 ms. In line with our previous findings, the mean β value was higher than zero in both groups, suggesting that at the group level there was no bias towards committing false-alarm errors. When inspecting individual performance, we observed two stroke patients who had performed at chance level. Both patients were unable to maintain fixation while seated due to motor limitations.

The frequency table depicted in Fig. 2 also shows that the distribution of performance seems to be somewhat skewed. Indeed, we calculated the skewness of the d’ parameter among the control group and we found a negative skew of −1.3 (SE=.374). Nevertheless, there is a possibility that this is a result of a relatively small sample size of aging adults, and this observation should be further tested with a larger sample. We encourage future studies to collect more data in order to better reflect the distribution among normal population. Such studies should also account for age-related changes in sustained attention that might influence our performance parameters. A negatively skewed distribution was also observed among patients (skewness = −1.5; SE = .409), although such an observation could be explained by the small, non-homogeneous, clinical sample. As opposed to the skewed distribution of the d’ parameter, the distribution of the bias (β) parameter was closer to symmetrical among controls (skewness = −.041; SE = .374) as well as among patients (skewness = −.243; SE = .409).

### Group comparisons

We compared the group detection rate (d’) and bias (β) using a t-test for independent samples (equality of variance not assumed). The group differed significantly in their d’ (t=3.082; *p*=.004; 95% CI [0.24–1.19]) with an overall higher detection rate among controls. The β did not differ between the groups (t=.234; p=.816). To make sure that the significant group difference is not driven by the two patients with performance at chance, we repeated the analysis excluding them. The results remained significant (t=2.756; *p*=.008; 95% CI [0.13–0.84]). These two comparisons show that the test can in principle differentiate a group of clinical participants from non-clinical, and that their overall pattern of performance is similar as reflected in the bias parameter.

## General discussion

We successfully managed to establish a new reliable method for increasing variability in accuracy in a CPT. By using a mask, we degraded the perceptual sensitivity to target (d’) and demonstrated how this parameter is correlated with a well-established construct of sustained attention – the RTSD. We also demonstrated that a change in the criteria parameter (β) characterizes the difference between a response inhibition task and a sustained attention task. Finally, we demonstrated the feasibility of the task for older adults and stroke survivors.

The increased sensitivity of the task is attributed to the use of a visual mask. According to our theoretical view, the use of masking increases the demand for attention. This view is in line with findings demonstrating that attention can reduce the effect of masking (Enns & Di Lollo, 1997), and enhance perception (e.g., Muller & Humphreys, 1991; Posner, 1980). Nevertheless, the deployment of visual masking also eliminates iconic memory representations in the process of encoding into VSTM (Smith, Ratcliff, & Wolfgang, 2004). In this respect, while the MCCPT paradigm aims to avoid load on memory components as in the case of the CPT-AX, there is a potential that the masking still increases working memory demands. Indeed, the exposure time used in our paradigm is significantly shorter than the mean reaction time, and therefore it is likely that the perceptual decision relies on the maintenance of the target in VSTM. However, in our view the involvement of memory mechanisms in the MCCPT does not overshadow the attentional requirements and is not comparable to the CPT-AX. First, while the CPT-AX requires the active maintenance of two objects in memory, the MCCPT requires only one item. In that respect, a memory capacity for a single item seems to be a prerequisite for nearly any visual discrimination task where participants need to remember a predefined target. Second, when it comes to the process of encoding the items from iconic memory to VSTM, we would argue that this is exactly where attention plays a major part: attention is the cognitive components which “transfer” visual objects into VSTM (e.g., Bundesen, 1990, 2005; Desimone & Duncan, 1995) within a much shorter timeframe (e.g., Vogel, Woodman, & Luck, 2006) than the one employed in the exposure times used in the MCCPT. In particular, the MCCPT relies on the visual presentation of repeating, overlearned, simple stimuli; in such cases, it is likely that early visual processes occur even faster due to the effects of learning (e.g., Ahissar & Hochstein, 1997). More compelling so, our empirical findings clearly support our view with the majority of our participants, even within the clinical group, adequately performing the task.

The current research has its own limitations. At this point, we highlighted how the different outcome measures respond to various parameters, and how they are related to each other. Nevertheless, there is still the question of the ecological validity of the task that should be further tested by trying to relate the task to how people can actually sustain their attention outside the laboratory settings (for example by looking into self-reported distractibility). Another possible limitation is related to the increased variability among healthy individuals performing the MCCPT. Potentially, a task that facilitates a higher error rate among non-impaired, might be too difficult for a clinical population. We recently presented an experimental study of the correlation between performance in our MCCPT task and self-reports of cognitive difficulties among chronic stroke patients and older adults where both populations performed a variation of the MCCPT with a relatively comparable, overall high performance (Shalev, Humphreys, & Demeyere, 2016). We now expand on this data in Experiment 3 where we demonstrated feasibility and distributions in this clinical population, evidencing that the task can be used (with a minor alternation of the exposure times) with target populations such as aging individuals and stroke survivors. We provide evidence that the MCCPT allows the assessment of performance on a CPT without relying on RTs. Nevertheless, one should keep in mind that some clinical populations at acute stages may not be able to perform a computerized cognitive task lasting 10 min.

Importantly, none of the task parameters should be set in stone: our main conclusion from Experiments 1 and 2 is that using a mask can be beneficial in increasing the number of errors in a CPT without interfering with the overall reliability. We also show that the target-distractor ratio should be considered carefully. Parameters such as SOAs, exposure times, and overall task length should be further manipulated in the future. Finally, future studies should focus on collecting data from larger samples of varying populations, in order to establish reliable normative data. While we acknowledge that the relatively small sample size we used in this study is an evident limitation, we believe that the converging evidence supports the use of the MCCPT as an alternative for standard CPT with a minimum exposure to confounds unrelated to attention.
